# A call to arms: on refining *Plasmodium vivax* microsatellite marker panels for comparing global diversity

**DOI:** 10.1186/1475-2875-12-447

**Published:** 2013-12-11

**Authors:** Patrick L Sutton

**Affiliations:** 1 Center for Genomics and Systems Biology, Department of Biology, New York University, 12 Waverly Place, New York, NY 10003, USA

**Keywords:** Malaria, Vivax, *Plasmodium*, Microsatellite, Genetic diversity

## Abstract

**Background:**

Microsatellite (MS) markers have become an important tool for studying the population diversity, evolutionary history and multiplicity of infection (MOI) of malaria parasite infections. MS are typically selected on the basis of being highly polymorphic. However, it is known that the polymorphic potential (mutability) of each marker can vary as much as two orders of magnitude, which radically changes how diversity is represented in the genome from one marker to the next. Over the past decade, approximately 240 *Plasmodium vivax* MS have been published, comprising nine major panels of markers. Inconsistent usage of each panel has resulted in a surfeit of descriptive genetic diversity data that are largely incomparable between populations. The objective of this study was to statistically evaluate the quality of individual MS markers in order to validate a refined panel of markers that will provide a balanced picture of *P. vivax* population diversity.

**Methods:**

All previously published data, including genetic diversity indices, MS parameters, and population parameters, were assembled from 18 different global studies into a flat file to facilitate statistical analysis and modelling using JMP® Genomics 6.0 (SAS Institute Inc, Cary, NC, USA). Statistical modeling was employed to down-select markers with extreme variation among the mean number of alleles, expected heterozygosity, maximum repeat length and/or chromosomal location of the repeat. Individual MS were analysed by step-down whole model linear regression and standard least squares fit models, both stratified by annual parasite incidence to identify MS markers with values significantly different from the mean.

**Results:**

Of the 42 MS under evaluation in this study, 18 (nine high priority) were identified as ideal candidates for measuring population diversity between global regions, while five (two high priority) additional markers were identified as candidates for MOI studies.

**Conclusions:**

MS diversity was found to be a function of endemicity and motif structure. Evaluation of individual MS permitted the assembly of a refined panel of markers that can be reliably utilized in the field to compare population structures between global regions.

## Background

Microsatellite (MS) DNA sequences are short tandem repeats, typically comprised of one (mono-) to six (hexa-) nucleotides (motifs), which repeat continuously without interruption (perfect repeat type), with intermittent nucleotide disruption (imperfect repeat type), with interrupting insertions (interrupted repeat type) or in tandem with a different motif (compound repeat type). MS are caused and maintained by mutation events, such as replication slippage and/or slip-strand mismatch repair, which induces sequence length variation through expansions/insertions and contractions/deletions of the repeating motif(s) [1-4]. Regardless of repeat type, the total number of repeats in the MS is referred to as the repeat length. Variation in the repeat length causes size polymorphisms within the locus, which can be used to differentiate organisms in population diversity studies [5,6]. Given their mechanisms of mutation, MS are often considered neutral. However, this is somewhat debated due to the fact that MS are scattered throughout intergenic and intragenic regions of most chromosomes; therefore, it is important to consider the location prior to data interpretation in an effort to subscribe to this neutral theory. Although MS lack the strain diversity resolution that whole genome sequencing provides, these markers remain an effective and easily deployable method for high-throughput genotyping in the field at moderate cost. Compared with single nucleotide polymorphism (SNP) genotyping, MS can provide increased resolution due to a higher polymorphic potential (i e, more alleles per locus), but can be problematic to interpret, standardize and calibrate across multiple studies.

Since the introduction of MS in population diversity studies, great insight has been gained into the amount of observed and expected genetic diversity within extant populations of eukaryotic parasites [7-16]. For malaria parasites, MS have rapidly become a popular alternative to polymorphic antigenic genes due to their purported neutrality, ubiquity throughout genomes and utility for describing the evolutionary history of global populations. Furthermore, the relatively unconstrained polymorphic nature of MS loci permits increased detection of multiclonal infections [17], which can be useful when describing the history of endemicity and the stability of transmission within a specific global region [18,19]. For *Plasmodium vivax*, the utility of these markers may even extend to describing infection dynamics across time, e g, whether an individual is presenting with a relapse, recrudescence or reinfection [12,20-22]. One of the major objectives in *Plasmodium* global diversity studies is to generate data that can be compared between populations of differing geographies, ecologies, climates, endemicities, and transmission intensities; however, such studies require standardizing experimental and analytical methods across a large and geographically separated community of researchers [23,24].

Unlike the frequently used *Plasmodium falciparum* MS marker panel published by Anderson *et al.*[8]*,* there are approximately nine different panels of *P. vivax* MS markers (including two panels with minisatellites with motifs that exceed six nucleotides) [12,25-32], describing at least 240 loci scattered throughout the genome. The majority of these MS markers were identified *in silico* and their polymorphic nature tested on DNA from reference strains [12,25-32]. However, in the last decade there have been at least 22 studies investigating *P. vivax* MS population diversity across seven global regions, 17 countries, and at 47 different field sites. Of the markers utilized in these studies (N = 68), only 42 have been tested in more than one field site, and seven of these are second-generation versions of a previously published marker, which results in moderately redundant population diversity data. Consequently, there are many sets of descriptive data that remain largely incomparable, owing to minimal genetic marker overlap between studies.

In most studies, microsatellites are selected on the basis of being highly polymorphic. However, it is known that the polymorphic potential of each marker can vary as much as two orders of magnitude, which radically changes how diversity is represented in the genome from one marker to the next [33-35]. The objective of this study was to statistically evaluate the quality of the MS markers currently in use, in order to generate a refined panel of markers that will provide a balanced picture of *P. vivax* genomic diversity. A statistically validated *P. vivax* MS panel would provide at least two benefits to the *P. vivax* community. First, statistical evaluation provides a means of assessing marker suitability at the outset of a study, for the purpose of describing population structure and multiplicity of infection (MOI). The inherent mutability of the repeat region is not easily assessed in the absence of long-term *in vitro* culture, which is not routine for *P. vivax* parasites due to their strict preference for reticulocytes. However, the quality of MS markers can be evaluated statistically by investigating the association between diversity level and endemicity, as well as, the repeat length [36-43], motif length, repeat type and location of the tandem repeat. The second benefit derived from the use of a standardized panel is the ability to compare population parameters, such as diversity and structure *between* global regions, which is a basic premise of population genetics studies. Before these benefits can be realized, the current MS marker panels must be re-evaluated and if possible consolidated to permit a more comprehensive and comparative approach to *P. vivax* population diversity across global populations.

Analyses resulted in a standardized panel of 18 (nine high priority) high-quality MS markers distributed across nine chromosomes. These markers are ideal for population diversity studies, as they will reliably describe overall population structure as a function of endemicity, while also accommodating a wide range of polymorphic variation. Additionally, a panel of five (two high priority) highly polymorphic MS markers was identified for MOI studies. These markers consistently exceed the predicted diversity level within different global regions and are suitable for describing infections with more than clone due to a possible increased mutability. Standardized usage of these panels will facilitate a clearer understanding of the history of this parasite as it has evolved in different ecological and epidemiological niches.

## Methods

### Microsatellite marker selection

Of the ~240 MS markers that have been described in the literature, 42 were selected for this study because each had been used in more than one field study, and therefore could be compared. These 42 MS markers were verified against the reference genomes [25,44], tested for redundancy against all published MS loci, located in the genome (intergenic or intragenic), and identified by repeat type (perfect or non-perfect, which includes all repeat types that are not deemed perfect). Of the 42 MS markers, seven were found to be second-generation versions of a previously published marker (first-generation), which had either been redesigned to optimally capture the repeat region or were unknowingly duplicated during the discovery stage (NCBI Primer Blast). In most studies, the second-generation marker was used in the same study as the first-generation marker, permitting a direct comparison among genetic diversity indices. In all cases, variation between first- and second-generation markers was insignificant. As a result, only data from the first-generation markers was utilized in this study, however, second-generation markers are identified throughout this manuscript in “( )” immediately following the first-generation name. Concatenating these multi-generation markers resulted in a final panel of 35 discrete MS markers. Further, genomic location with respect to presence within intergenic or intragenic regions was determined. Of the 35 markers, 20 were located in known or hypothetical genes, while only 15 were located in non-coding intergenic regions. The repeat type also varied, with 26 MS markers identified as having perfect repeats and nine with non-perfect repeats. Additional file 1 describes each of the MS loci analysed in this study.

### Data consolidation

All previously published data, including genetic diversity indices (ie, number of alleles per locus and expected heterozygosity (H_e_) and repeat length size), MS parameters (ie, location, repeat type, and motif length) and population parameters (ie, regional location, annual parasite incidence (API) and sample sizes), were assembled from 18 different global studies (representing seven regions, 14 countries, and 35 field sites) into a single database to facilitate statistical analysis and modelling using JMP® Genomics 6.0 (©2012 SAS Institute Inc, Cary, NC, USA). See Additional file 2 for a summary of studies included in the analysis.

Given the fact that genetic diversity is a function of endemicity, it was essential to establish endemicity categories to stratify downstream analyses. However, the reported metrics for calculating malaria incidence varied extensively across the global regions examined in this study. In an effort to accommodate this variation, all metrics were simplified by converting them to the “annual parasite incidence” (API - the number of microscopically confirmed malaria cases during one year per 1,000) during the time at which the samples were collected for each study. Previously described methods for classifying endemicity [45] were utilized to permit categorical transformation of the numerical API values (≤0.05 stratum, hypo-endemic and typically focal transmission; >0.05 stratum, meso- to hyper-endemic) to facilitate data analysis.

### Defining the polymorphic potential of individual MS

**Figure 1:**
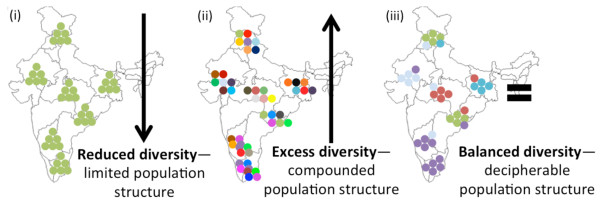
**Population structure scenarios based on the polymorphic potential of microsatellite markers. (i)** Markers generating *reduced diversity* due to reduced mutability will not be able to resolve existing population structure; **(ii)** markers generating *excess diversity* due to increased mutability may confound local population structure, making it difficult to compare different geographic regions; **(iii)** markers with *balanced diversity,* calibrated to the population comparisons of interest, can decipher population structure and provide meaningful insight into parasite migration and evolution.

**Figure 2:**
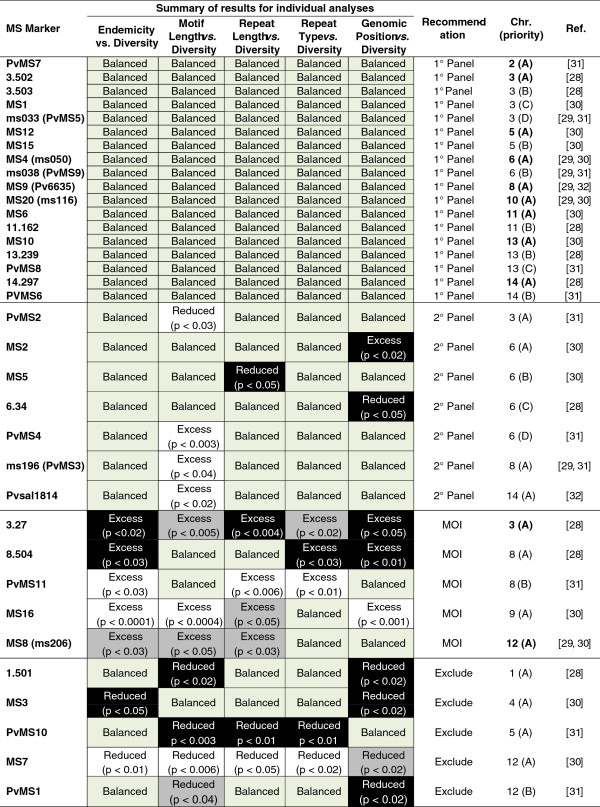
**Summary of statistically validated *P. vivax *MS for usage in population diversity and MOI studies.** MS diversity indices (mean number of alleles per locus, expected heterozygosity, and maximum repeat length) were correlated with six microsatellite and population parameters (endemicity, motif length, repeat length, repeat type and genomic position) to identify MS with excess, reduced or balanced diversity in comparison with the mean. Balanced in both API categories (API >0.05 or API ≤0.05) is indicated in green. Excess or reduced in API >0.05, API ≤0.05 or both is indicated in white, black and gray, respectively. Based on this data, MS were categorized into four recommended groups: 1° Panel, 2° Panel, Exclude, and MOI. “1° Panel” indicates balanced diversity in all six test categories and usage as the primary panel of markers for measuring population diversity. “2° Panel” indicates markers with significant excess or reduction of diversity in one of six test categories. These markers should be used cautiously, as they may misrepresent the diversity level due to inherent unbalanced mutability. “Exclude” indicates markers with significant reduction in diversity in more than one of the six test categories. These markers are not recommended, as they consistently result in a misrepresentation of population diversity due to reduced polymorphic potential. “MOI” (multiplicity of infection) indicates MS markers that consistently have significant excess diversity in more than one test category. MOI markers are ideal for identifying multiclonal infections. For chromosomes with more than one MS marker tested, priority has been assigned (A-D). Priority is based on the total number of studies that have utilized the marker, with a higher priority being placed on markers that have been used more frequently. Bold font indicates markers of highest priority.

The objective of this study was to identify quality MS markers in order to generate a refined panel of markers that will provide a balanced picture of *P. vivax* genomic diversity. Given the fact that the polymorphic potential of each marker can generate unequal variation [33-35], statistical modelling was employed to down-select markers with extreme variation. Number of alleles, expected heterozygosity (H_e_) and/or repeat lengths in excess of the mean may indicate unregulated polymorphic potential, with heightened heterogeneity that can obscure downstream population parameter estimations (Figure 1). Although MS markers in excess of the mean may not directly translate into distinct and observable patterns within the parasite population structure, these markers can be used as tools to define the MOI. Conversely, a reduction of alleles, H_e_, and/or repeat length may not provide a strong enough signal to discern population structure when it does exist (Figure 1). Though no studies have indicated an overall reduction in MS diversity, this is expected to become more of a concern in regions with elimination platforms, as diversity decreases with reduced transmission. Markers in significant excess of the mean are termed “Excess”, those significantly reduced from the mean are termed “Reduced”, and those with no difference from the mean are termed “Balanced”. For these reasons, markers that deviate significantly from the mean in either direction were down-selected from the final core panel of markers, which can be used to clearly define the population structure without bias from excess or reduced diversity (Figure 2). In all cases, individual MS were analysed in step-down whole model linear regression and standard least squares fit models, both stratified by API, to identify markers with values significantly different from the mean.

## Results and discussion

### MS diversity as a function of endemicity

**Figure 3:**
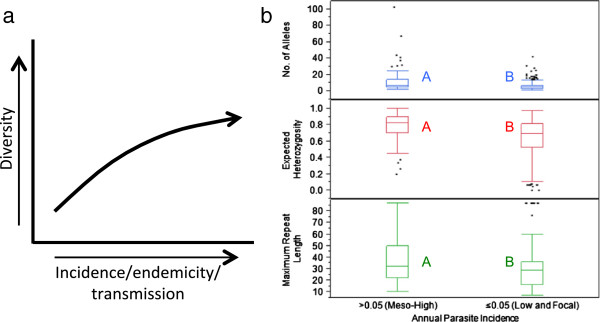
**a, b MS diversity is a function of parasite endemicity. (a)** Schematic illustrating that population diversity increases as a function of increased endemicity, captured by parasite incidence and transmission; **(b)** for all MS markers combined, the box plots compare the mean number (no) of alleles per locus (y-axis, blue), expected heterozygosity (H_e_) (y-axis, red) and maximum repeat length (y-axis, green) between different API categories (x-axis). Character values (A and B) denote statistical significance between API strata (p <0.0001, ANOVA).

The amount of genetic diversity within a region is a function of parasite incidence [46-48], and high quality MS markers should reflect this relationship (Figure 3a). To test the overall link between diversity and endemicity, diversity indices for all MS markers across all global studies were correlated with the categorical API strata. For the API >0.05 stratum, the mean number of alleles per locus ( $\bar{x}$ = 11.4, σ = 11.9, 95% CI = 9.4, 13.4), mean H_e_ ( $\bar{x}$ = 0.79, σ = 0.15, 95% CI = 0.76, 0.81) and the mean maximum repeat length ( $\bar{x}$ = 36.3, σ = 19.8, 95% CI = 33.0, 40.0) was significantly higher than the mean number of alleles per locus ( $\bar{x}$ = 6.7, σ = 5.4, 95% CI = 6.0, 7.4), mean H_e_ ( $\bar{x}$ = 0.63, σ = 0.24, 95% CI = 0.60, 0.66) and the mean maximum repeat length ( $\bar{x}$ = 31.8, σ = 18.9, 95% CI = 29.4, 34.2) in the API ≤0.05 stratum (p <0.0001, p <0.0001 and p = 0.0294, ANOVA, respectively) (Figure 3b). This confirms that genetic diversity is a function of parasite endemicity, as regions with greater endemicity are expected to have a greater repertoire of genetically diverse parasites circulating in the population. Individual analysis for each MS, including down-selection data and panel recommendations, can be found in Figure 2.

### Polymorphic potential of repetitive regions

#### Understanding the role of microsatellite parameters on diversity

Earlier reports considering the polymorphic potential of *P. vivax* MS identified differences in motif length and repeat length as likely causes for allelic variation between MS markers [37,41,43]; however, much of this discussion was had prior to the publication of the draft genome [25] and subsequent whole genome sequencing projects [44,49]. In other organisms, like fruit flies, humans and chimpanzees, researchers have found that certain motifs, based on their length and nucleotide composition, have higher rates of mutability than others, suggesting that repeat length is an intrinsic function of motif mutability [36,38-40]. However, this circularity of this hypothesis is difficult to break and one cannot help but question the root cause for increased mutability, as the size of the repeat must in part be a of the function of motif mutability. Here, both motif length and repeat length are re-investigated, as well as, the genomic location of the tandem repeat (intergenic *versus* intragenic) and the repeat type (perfect, interrupted, compound or interrupted and compound) as likely factors for MS mutability.

### Motif length as a function of MS diversity

**Figure 4:**
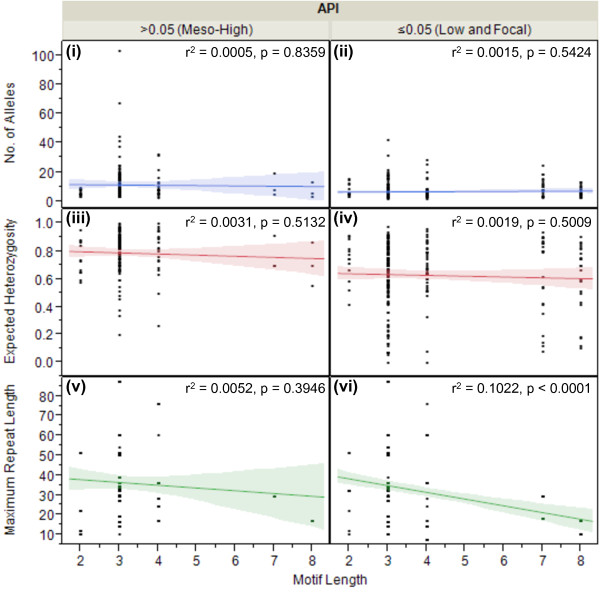
**Motif length as a function of MS diversity.** For all MS markers combined, the box plots compare the mean number (no) of alleles per locus (y-axis, blue), expected heterozygosity (H_e_) (y-axis, red) and maximum repeat length (y-axis, green) across all motif lengths (x^1^°-axis), stratified API (x^2^°-axis). There were no significant correlations between motif length and the mean no of alleles per locus **(panels i, ii)** or mean H_e_**(panels iii, iv)** in either API stratum. However, in the ≤0.05 API stratum there was a significant negative correlation between motif length and mean maximum repeat length **(panel vi)** (p <0.0001, ANOVA, bivariate fit). This correlation exists only as a trend for the >0.05 API stratum **(panel v)** (p = 0.349, ANOVA, bivariate fit).

The 35 markers included in this study displayed five different motif lengths: di- (n = 2), tri- (n = 18), tetra- (n = 8), hepta- (n = 2), and octa- (n = 2) nucleotide. Though the hepta- and octa-nucleotide motifs are not true microsatellites, but rather minisatellites, the use of these markers in more than one field site warrants consideration in this analysis. Of these five motif lengths, tri-nucleotide motifs revealed the most dynamic range of polymorphic potential, with the largest range of alleles (range = 1–103), H_e_ (range = 0.01-0.99) and maximum repeat length (range = 10–87). Octa-nucleotide motifs revealed the most conservative polymorphic potential, with the smallest range of alleles (range = 2–13), H_e_ (range = 0.01-0.9) and maximum repeat length (range = 10–17) (Figure 4); though an increased sample size is required to have adequate power to be confident in this result.

Next, a linear regression was used to determine the relationship between motif length and the mean number of alleles per locus, H_e_ and maximum repeat length for all MS, stratified by API. There were no significant correlations between motif length and the mean number of alleles per locus or H_e_ in either API stratum (Figure 4). However, in the ≤0.05 API stratum there was a significant negative correlation between motif length and mean maximum repeat length (p <0.0001, ANOVA, bivariate fit), suggesting that shorter motif lengths may generate an increased number of repeats; however, this is not reflected in the number of alleles per locus (Figure 4). This correlation exists only as a trend for the >0.05 API stratum (p = 0.349, ANOVA, bivariate fit), likely due the limited usage of hepta- and octa-nucleotide motifs in regions of higher endemicity (Figure 4). Regardless, the negative correlation between motif length and repeat length establishes the motif structure as an important factor to be considered when selecting MS markers for genetic diversity studies. Individual analysis for each MS, including down-selection data and panel recommendations, can be found in Figure 2.

### Repeat length as a function of MS diversity

**Figure 5:**
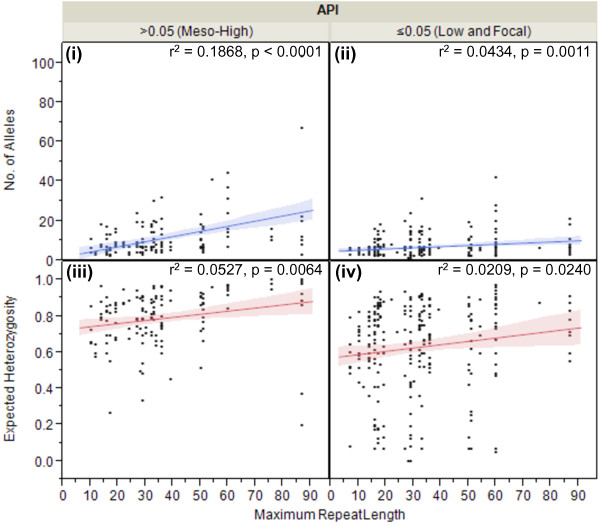
**Repeat length as a function of MS diversity.** For all MS markers combined, the box plots compare the mean number (no) of alleles per locus (y-axis, blue) and expected heterozygosity (H_e_) (y-axis, red) across repeat length (x^1^°-axis), stratified API (x^2^°-axis). In both API strata, API ≤0.05 **(panels ii, iv)** and API >0.05 **(panels i, iii)**, the mean no of alleles per locus (p = 0.0011 and p = 0.0240, ANOVA, bivariate fit, respectively) and mean H_e_ (p <0.0001 and p = 0.0064, ANOVA, bivariate fit, respectively) were positively correlated with the repeat length.

Previous studies have reported that the mutability of the repeat region may be guided by the repeat length, as increased replication slippage is probable in sequences with high repeat numbers [21,38,41-43]. In this study, repeat length was highly variable, ranging from seven to 87 repeats across the 35 MS markers. Statistical modelling was used to correlate the mean number of alleles per locus and H_e_ with the mean maximum number of repeats in the repeat length array across all studies, stratified by API. In both API strata, API ≤0.05 and API >0.05, there was a significant positive linear correlation between the number of alleles per locus (p = 0.0011 and p = 0.0240, ANOVA, bivariate fit, respectively) and H_e_ (p <0.0001 and p = 0.0064, ANOVA, bivariate fit, respectively) with increasing repeat length (Figure 5). These results confirm previous work by Russell *et al.*[37] and provide additional insight into the maintenance of tandem repeats, as the parasites are transmitted with different rates in regions of differing endemicity. Individual analysis for each MS, including down-selection data and panel recommendations, can be found in Figure 2.

### Repeat type as a function of MS diversity

Sequence analysis of MS loci has revealed that MS may exist in either perfect or non-perfect types. Perfect microsatellites will have a repeated motif that continues uninterrupted for a specific repeat length, while non-perfect microsatellites may exist as imperfect, interrupted or compound repeats. Although hard evidence is lacking for the cause of these non-perfect repeat types, the generation of single point mutations within a MS motif may offer some explanation for imperfect repeats, while interrupted repeat types may be caused by insertion mutations and compound repeat types may be the result of recombinatory events. Regardless of the mechanistic cause, the mutability of these different repeat types is of considerable interest as it may assist in the selection of quality MS loci for population diversity studies. As mentioned in the Methods section, of the 35 markers examined in this study, 26 MS markers were identified as having perfect repeats and nine were defined as non-perfect (either imperfect, interrupted or compound). For the purpose of this analysis the repeat type, limited to perfect *versus* non-perfect repeat types, was correlated with the mean number of alleles per locus, H_e_ and maximum repeat length (stratified by API).

**Figure 6:**
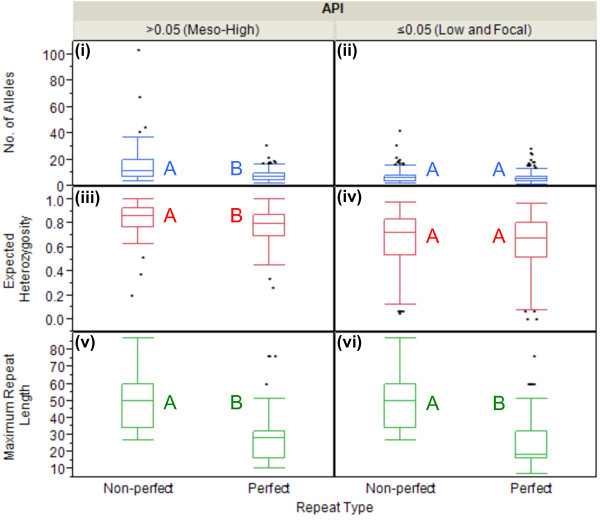
**Repeat type as a function of MS diversity.** For all MS markers combined, the box plots compare the mean number (no) of alleles per locus (y-axis, blue), expected heterozygosity (H_e_) (y-axis, red) and maximum repeat length (y-axis, green) across different repeat types (x^1^°-axis), stratified API (x^2^°-axis). Character values (A and B) denote statistical significance between perfect and non-perfect repeat types, within each API category. In the API >0.05 stratum, non-perfect repeat types had a significantly higher mean no of alleles per locus **(panel i)** (p <0.0001, ANOVA), mean H_e_**(panel iii)** (p = 0.0192, ANOVA) and mean maximum repeat length **(panel v)** (p <0.0001, ANOVA) than perfect repeat types. In the API ≤0.05 stratum, non-perfect repeat types had a significantly higher mean maximum repeat length **(panel vi)** (p <0.0001, ANOVA) than perfect repeat types. No significant differences were found between repeat types for either the mean no of alleles per locus **(panel ii)** or mean H_e_ in the API ≤0.05 stratum **(panel iv)**.

On the most basic level, non-perfect repeat types appear to be associated with increased diversity in all diversity indices, regardless of API stratification. In the API ≤0.05 stratum, the mean maximum repeat length for the non-perfect repeats ( $\bar{x}$ = 48.3, σ = ±17.7, 95% CI = 44.3, 52.3) was significantly higher than the mean for perfect repeats ( $\bar{x}$ = 23.9, σ = ±13.6, 95% CI = 22.0, 26.0) (p <0.0001, ANOVA) (Figure 6). A similar observation was made in the API >0.05 stratum, where the mean maximum repeat length for the non-perfect repeats ( $\bar{x}$ = 49.6, σ = ±19.3, 95% CI = 44.5, 54.8) was also significantly higher, compared with perfect repeats ( $\bar{x}$ = 27.1, σ = ±14.1, 95% CI = 24.0, 30.2) (p <0.0001, ANOVA) (Figure 6). Significance between these repeat types was also achieved when considering the mean number of alleles per locus and the H_e_ in the API >0.05 stratum. Non-perfect repeats had a significantly higher mean number of alleles per locus ( $\bar{x}$ = 16.2, σ = ±16.5, 95% CI = 11.8, 20.6) and H_e_ ( $\bar{x}$ = 0.82, σ = ±0.15, 95% CI = 0.78, 0.86), when compared with the mean number of alleles per locus ( $\bar{x}$ = 8.1, σ = ±5.2, 95% CI = 6.9, 9.2) (p <0.0001, ANOVA) and H_e_ ( $\bar{x}$ = 0.77, σ = ±0.14, 95% CI = 0.73, 0.80) (p = 0.0192, ANOVA) of the perfect repeats (Figure 6).

However, it seems counterintuitive that non-perfect repeats might generate increased diversity levels in these populations. Further investigation of these repeat types revealed that when compared with perfect repeats ( $\bar{x}$ = 4.0, σ = ±1.9, 95% CI = 3.8, 4.2), non-perfect repeats ( $\bar{x}$ = 3.1, σ = ±0.31, 95% CI = 3.1, 3.2) are significantly biased towards smaller motif lengths (p <0.0001, ANOVA), which were previously found to be associated with increased diversity levels in the population. Therefore, it is more likely that the increased diversity found to be associated with non-perfect repeats is a byproduct of the actual motif structure or the combination of different repeating motifs when the non-perfect repeat is a compound type. Individual analysis for each MS, including down-selection data and panel recommendations, can be found in Figure 2.

### Genomic position as a function of MS diversity

The proximity of a MS to a coding region in the genome will likely influence the polymorphic potential within the locus. For example, recent studies in *P. falciparum* have indicated that H_e_ in MS is inversely correlated with the proximity of the MS locus to the *P. falciparum* chloroquine resistance transporter gene, which is known to be associated with chloroquine resistance in this parasite [50-53]. This relationship is likely a result of genetic hitchhiking, but is still important to consider when selecting MS loci to describe genetic diversity in a population as it may impact the polymorphic potential. As previously mentioned, of the 35 markers examined in this study, 20 were located in known (N = 8) or hypothetical genes (N = 12), while only 15 were located in non-coding intergenic regions. For the purpose of this analysis the genomic position was correlated with the mean number of alleles per locus, H_e_ and maximum repeat length (stratified by API).

**Figure 7:**
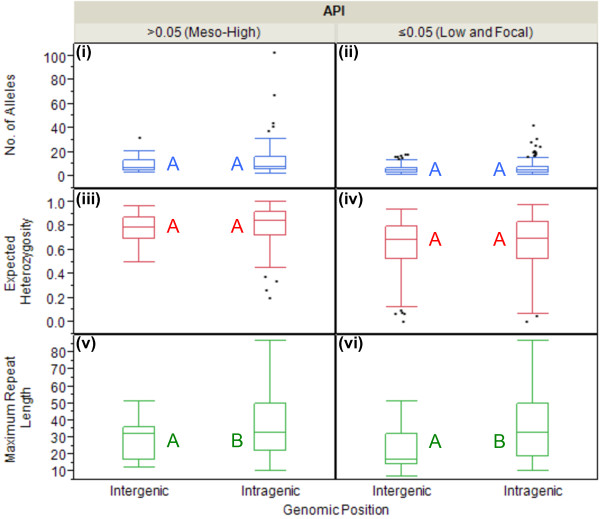
**Genomic position as a function of MS diversity.** For all MS markers combined, the box plots compare the mean number (no) of alleles per locus (y-axis, blue), expected heterozygosity (H_e_) (y-axis, red) and maximum repeat length (y-axis, green) across different genomic positions (x^1^°-axis), stratified API (x^2^°-axis). Character values (A and B) denote statistical significance between intergenic and intragenic locations, within each API category. For the both API strata, there were no significant differences among the mean no of alleles **(panels i, ii)** per locus or mean H_e_**(panels iii, iv)** between intergenic and intragenic regions. In both API strata, API ≤0.05 **(panel vi)** and API >0.05 **(panel v)**, the mean maximum repeat length did vary significantly between intergenic and intragenic, with intragenic loci having significantly higher numbers of repeats than intergenic regions (p <0.0001 and p = 0.0303 for API ≤0.05 and API >0.05, respectively, ANOVA).

For the both API strata, there were no significant differences among the mean number of alleles per locus or mean H_e_ between intergenic and intragenic regions. However, in both API strata, API ≤0.05 and API >0.05, the mean maximum repeat length did vary significantly between intergenic ( $\bar{x}$ = 24.0, σ = ±13.5, 95% CI = 21.2, 26.7; $\bar{x}$ = 31.1, σ = ±13.0, 95% CI = 27.2, 35.0) and intragenic ( $\bar{x}$ = 36.8, σ = ±20.1, 95% CI = 33.6, 40.1; $\bar{x}$ = 38.8, σ = ±22.0, 95% CI = 34.2, 43.3, respectively), with intragenic loci having significantly higher numbers of repeats than intergenic regions (p <0.0001 and p = 0.0303 for API ≤0.05 and API >0.05, respectively, ANOVA) (Figure 7). To help explain this finding, genomic position was correlated with repeat type (perfect *versus* non-perfect) and motif length. In this study, intragenic MS are significantly comprised of non-perfect repeat types compared with the intergenic MS, 44.1% compared with 23.3%, respectively (p <0.0001, Fisher’s Exact). Likewise, these highly diverse intragenic markers are significantly biased towards smaller motif lengths (p <0.0001, ANOVA). Neglecting to observe an increase in the mean number of alleles or H_e_, would likely negate the possibility that these intragenic regions have increased polymorphic potential, but again, this analysis revealed that there is a fundamental association between the structure of the motif and the amount of genetic diversity present in the MS. Individual analysis for each MS, including down-selection data and panel recommendations, can be found in Figure 2.

## Conclusions

Genetic diversity data were mined from 18 population diversity studies (Additional file 1) in an effort to evaluate the quality of data generated from published *P. vivax* MS markers (N = 42, reduced to N = 35 after NCBI Primer Blast indicated redundancies) and also to produce recommended MS panels for both population diversity and MOI studies. Though there is a convention among population diversity studies to select MS markers with extremely high polymorphic potential, there are MS with increased and decreased mutation rates that will falsely inflate and deflate the genetic diversity of parasite population, respectively. Therefore, when considering individual MS, markers may generate excess, reduced or balanced (no difference) diversity when compared with the mean across all markers (Figure 1). Given the inherent unequal MS mutability [33-35], data quality was examined by using robust step-down statistical models that compared the genetic diversity metrics (number of alleles per locus, H_e_ and maximum repeat lengths) of individual MS markers with the mean of all MS markers (stratified by API) to examine the impact of parasite endemicity, motif length, repeat length, repeat type and genomic position as a function of MS diversity. Individual analysis for each MS, including down-selection data and panel recommendations, can be found in Figure 2.

As expected, the results indicated that the amount of genetic diversity present within all global regions is a function of parasite endemicity; individual MS analysis revealed that five of the 35 markers were in significant excess of the mean, while two were significantly reduced from the mean (Figure 2, Figure 3a,b). Other factors, such as the motif length and repeat length, were also significantly correlated with the amount of diversity present within individual MS markers. Compared with longer motifs, shorter motifs were associated with increased genetic diversity; six MS markers were in significant excess of the mean, while five were significantly reduced (Figure 2, Figure 4). Longer repeat lengths, rather than shorter repeat lengths, were positively correlated with greater diversity; four of the MS markers were in significant excess of the mean, while three were significantly reduced (Figure 2, Figure 5). Further, non-perfect repeats and intragenic MS also correlated significantly with increased genetic diversity. For repeat type, there were three MS in significant excess of the mean and two significantly reduced from the mean (Figure 2, Figure 6); while MS location revealed four in significant excess of the mean and five significantly reduced from the mean (Figure 2, Figure 7). However, non-perfect repeat types were biased towards being located within intragenic regions and shorter motifs with longer repeat lengths tended to comprise both non-perfect repeats and intragenic MS. Therefore, it is difficult to completely resolve the total impact of these MS parameters on genetic diversity.

The availability of a validated refined panel of MS markers will greatly facilitate the development of improved comparative population genetics algorithms, which will in turn generate a better understanding of the migration and evolution of this species. Based on the analyses in this study, MS markers have been categorized into four groups: (1) 1° Panel, (2) 2° Panel, (3) Excluded and (4) MOI (Figure 2). For chromosomes with more than one MS marker tested, a priority ranking has been assigned (A-D) (Figure 2). Priority is based on the total number of studies that have utilized the marker, with a higher priority being placed on markers that have been used more frequently. “1° Panel” (N = 18) indicates balanced diversity in all test categories and usage as the primary panel of markers for decoding population diversity and structure. It is recommended that future studies utilize MS markers with “A” priority ranking (N = 9, bold font) to facilitate population diversity comparison between global regions, as these markers have previously been used with the highest frequency. “2° Panel” (N = 7) indicates significant excess or reduction in diversity in one test category. It is recommended that the 2° Panel markers be used cautiously as additional markers to the 1° Panel, as the resulting population structure may be skewed towards decreased or increased diversity due to the inherent unbalanced mutability of the MS marker. “Exclude” (N = 5) indicates significant reduction in diversity in more than one test category. If selected for a population diversity study, it is recommended that the data be thoroughly scrutinized, as these markers will result in a skewed interpretation of population diversity due to the reduced polymorphic potential of these MS markers. “MOI” (N = 5) indicates MS markers that consistently have significant excess diversity in more than one test category. MOI markers are highly recommended for identifying multiclonal infections. Two of these five MS markers (3.27 and MS8 (ms206), bold font) are highly recommended for MOI studies due to having extreme excess diversity in more than one test category across both API strata.

## Competing interests

The author has declared no competing interests.

## Author’s contribution

PLS conceived idea, developed the design, acquired the data, performed data analysis and wrote the manuscript.

## Supplementary Material

Additional file 1 Description of microsatellite markers analyses. Click here for file

Additional file 2 ***Plasmodium vivax *** **microsatellite marker panels used in global studies.** Click here for file
