# Supplementary material for: A call to arms: on refining Plasmodium vivax microsatellite marker panels for comparing global diversity
Source: Malar J. 2013 Dec 11;12:447. doi: 10.1186/1475-2875-12-447 (PMC3878832; doi:10.1186/1475-2875-12-447)
Supplement: Additional file 1 — Description of microsatellite markers analyses. [file 1475-2875-12-447-S1.doc]

**Additional File 1**. Description of microsatellite markers analyzed

| **Reference** | **Microsatellite name**  **(2nd generation)** | **Chr.** | **Motif** | **Motif length** | **Fragment size** | **Location** | **Intergenic / Intragenic** | **Repeat type** |
| --- | --- | --- | --- | --- | --- | --- | --- | --- |
|  | 1.501 | 1 | GGTGAGA | 7 | 174 | 473731-473905 | Intragenic | Perfect |
|  | 3.27 | 3 | AAAC | 4 | 211 | 493109-493320 | Intragenic | Perfect |
|  | 3.502 | 3 | AACGGATG | 8 | 188 | 451078-451266 | Intergenic | Perfect |
|  | 3.503 | 3 | AAAAAGGC | 8 | 246 | 664880-665126 | Intergenic | Perfect |
|  | 6.34 | 6 | AC | 2 | 211 | 612809-613020 | Intergenic | Perfect |
|  | 8.504 | 8 | TGACCAA | 7 | 286 | 1324396-1324682 | Intergenic | Perfect |
|  | 11.162 | 11 | ATAC | 4 | 246 | 1859236-1859482 | Intergenic | Perfect |
|  | 13.239 | 13 | TTTA | 4 | 210 | 1601740-1601950 | Intergenic | Perfect |
|  | 14.297 | 14 | AAG | 3 | 228 | 2965457-2965685 | Intragenic | Perfect |
|  | MS1 | 3 | GAA | 3 | 235 | 451787-452022 | Intergenic | Perfect |
|  | MS2 | 6 | (TAAA)_2_TATA(TAAA)_6_TATA(TAAA)_19_ | 4 | 250 | 256393-256643 | Intergenic | Compound |
|  | MS3 | 4 | GAA | 3 | 189 | 296580-296769 | Intragenic | Perfect |
|  | MS4 (ms050) | 6 | AGT | 3 | 220 | 390260-390480 | Intergenic | Perfect |
|  | MS5 | 6 | CCTCTT(CCT)11 | 3 | 174 | 538573-538747 | Intergenic | Compound |
|  | MS6 | 11 | (TCC)_2_(TCT)_3_(CCT)_2_(TCC)_2_GCTTCT(TCC)_10_ | 3 | 241 | 1760679-1760920 | Intragenic | Interrupted / Compound |
|  | MS7 | 12 | GAA | 3 | 151 | 1185287-1185438 | Intragenic | Perfect |
|  | MS8 (ms206) | 12 | (CAG)_2_(CAA)_11_ | 3 | 198 | 2322322-2322520 | Intragenic | Compound |
|  | MS9 (Pv6635) | 8 | GGA | 3 | 188 | 1394222-1394410 | Intragenic | Perfect |
|  | MS10 | 13 | GAA(GGA)_2_AGA(GGA)_9_AGA(GGA)_4_AGAGGAAGA(GGA)_3_AGAGGAAGA(GGAAAA)_4_(GGA)_2_(AGA)_11_(GGA)_3_ (AGA)_2_GGAAGA(GGA)_2_ | 3 | 249 | 276454-276703 | Intragenic | Interrupted / Compound |
|  | MS12 | 5 | (TTC)_10_(TGC)_4_ | 3 | 220 | 70293-70513 | Intragenic | Compound |
|  | MS15 | 5 | TCT | 3 | 243 | 1110659-1110902 | Intragenic | Perfect |
|  | MS16 | 9 | (ACA)_9_GCA(ACA)_3_GCA(ACA)_7_GCA(ACA)_3_GCAATC(ACA)_2_ACC(ACA)_4_ACC(ACA)_3_GCAATC(ACA)_13_ | 3 | 238 | 1593215-1593453 | Intragenic | Interrupted / Compound |
|  | MS20 (ms116) | 10 | (GAA)_11_GAG(GAA)_13_(CAA)_4_GAA(CAA)_5_ | 3 | 211 | 1345593-1345804 | Intragenic | Interrupted / Compound |
|  | ms033 (PvMS5) | 3 | CAT | 3 | 147 | 30375-30522 | Intragenic | Perfect |
|  | ms038 (PvMS9) | 6 | CATA | 4 | 217 | 957019-957236 | Intragenic | Perfect |
|  | ms196 (PvMS3) | 8 | AT | 2 | 220 | 1631108-1631328 |  | Perfect |
|  | PvMS1 | 12 | GT | 2 | 246 | 2075886-2076132 |  |  |
|  | PvMS2 | 3 | CA | 2 | 302 | 782946-783248 | Intragenic | Perfect |
|  | PvMS4 | 6 | TA | 2 | 155 | 1027328-1027483 | Intragenic | Perfect |
|  | PvMS6 | 14 | TGA | 3 | 237 | 2835596-2835833 | Intragenic | Perfect |
|  | PvMS7 | 2 | TAA | 3 | 395 | 152238-152633 | Intergenic | Perfect |
|  | PvMS8 | 13 | TGTA | 4 | 314 | 1561307-1561621 | Intergenic | Perfect |
|  | PvMS10 | 5 | TATG | 4 | 301 | 1170542-1170843 | Intergenic | Perfect |
|  | PvMS11 | 8 | CATA | 4 | 374 | 840625-840999 | Intragenic | Perfect |
|  | Pvsal1814 | 14 | AGA | 3 | 652 | 2997128-2997780 | Intragenic | Interrupted / Compound |

* Microsatellite fragment sizes were calculated *in silico* using the reference genomes .

**References**

1. Imwong M, Nair S, Pukrittayakamee S, Sudimack D, Williams JT, Mayxay M, Newton PN, Kim JR, Nandy A, Osorio L , Carlton JM, White NJ, Day NP, Anderson TJ: **Contrasting genetic structure in Plasmodium vivax populations from Asia and South America.** *Int J Parasitol* 2007, **37:**1013-1022.

2. Karunaweera ND, Ferreira MU, Hartl DL, Wirth DF: **Fourteen polymorphic microsatellite DNA markers for the human malaria parasite Plasmodium vivax.** *Molecular Ecology Notes* 2007, **7:**172-175.

3. Joy DA, Gonzalez-Ceron L, Carlton JM, Gueye A, Fay M, McCutchan TF, Su XZ: **Local adaptation and vector-mediated population structure in Plasmodium vivax malaria.** *Mol Biol Evol* 2008, **25:**1245-1252.

4. Rezende AM, Tarazona-Santos E, Fontes CJ, Souza JM, Couto AD, Carvalho LH, Brito CF: **Microsatellite loci: determining the genetic variability of Plasmodium vivax.** *Trop Med Int Health* 2010, **15:**718-726.

5. Van den Eede P, Erhart A, Van der Auwera G, Van Overmeir C, Thang ND, Hung le X, Anne J, D'Alessandro U: **High complexity of Plasmodium vivax infections in symptomatic patients from a rural community in central Vietnam detected by microsatellite genotyping.** *Am J Trop Med Hyg* 2010, **82:**223-227.

6. Carlton JM, Adams JH, Silva JC, Bidwell SL, Lorenzi H, Caler E, Crabtree J, Angiuoli SV, Merino EF, Amedeo P, Cheng Q, Coulson RM, Crabb BS, Del Portillo HA, Essien K, Feldblyum TV, Fernandez-Becerra C, Gilson PR, Gueye AH, Guo X, Kang'a S, Kooij TW, Korsinczky M, Meyer EV, Nene V, Paulsen I, White O, Ralph SA, Ren Q, Sargeant TJ, Salzberg SL, Stoeckert CJ, Sullivan SA, Yamamoto MM, Hoffman SL, Wortman JR, Gardner MJ, Galinski MR, Barnwell JW, Fraser-Liggett CM: **Comparative genomics of the neglected human malaria parasite Plasmodium vivax.** *Nature* 2008, **455:**757-763.

7. Neafsey DE, Galinsky K, Jiang RH, Young L, Sykes SM, Saif S, Gujja S, Goldberg JM, Young S, Zeng Q, Chapman SB, Dash AP, Anvikar AR, Sutton PL, Birren BW, Escalante AA, Barnwell JW, Carlton JM: **The malaria parasite Plasmodium vivax exhibits greater genetic diversity than Plasmodium falciparum.** *Nat Genet* 2012, **44:**1046-1050.
