# Supplementary material for: A call to arms: on refining Plasmodium vivax microsatellite marker panels for comparing global diversity
Source: Malar J. 2013 Dec 11;12:447. doi: 10.1186/1475-2875-12-447 (PMC3878832; doi:10.1186/1475-2875-12-447)
Supplement: Additional file 2 — Plasmodium vivax microsatellite marker panels used in global studies. [file 1475-2875-12-447-S2.doc]

**Additional File 2. *Plasmodium vivax* microsatellite marker panels used in global studies**

| **Reference** | **Region** | **Country (field site)*** | **Marker Family^§^** | **No. of MS markers^†^** | **API** | **Sample size** |
| --- | --- | --- | --- | --- | --- | --- |
|  | South America | Brazil (a) |  | 14 | >0.05 | 25 |
|  |  | Brazil (b) |  |  |  | 50 |
|  | Africa | Ethiopia |  | 12 | >0.05 | 118 |
|  | Southern Asia | Myanmar |  |  |  | 167 |
|  |  | Sri Lanka |  |  | ≤0.05 | 140 |
|  | Southeast Asia | Republic of Korea (a) |  | 4 | ≤0.05 | 29 |
|  |  | Republic of Korea (a) |  | 9 |  | 29 |
|  |  | Republic of Korea (b) |  | 4 |  | 58 |
|  |  | Republic of Korea (b) |  | 9 |  | 58 |
|  | South America | Colombia (a) |  | 9 | ≤0.05 | 15 |
|  |  | Colombia (b) |  |  |  | 19 |
|  |  | Colombia (c) |  |  |  | 15 |
|  |  | Colombia (d) |  |  |  | 9 |
|  |  | Colombia (e) |  |  |  | 12 |
|  | Southeast Asia | India |  |  |  | 67 |
|  | Southern Asia | Laos |  |  |  | 71 |
|  |  | Thailand |  |  |  | 88 |
|  | Southern Asia | India |  | 3 | ≤0.05 | 38 |
|  |  | Myanmar |  |  |  | 75 |
|  |  | Thailand |  |  |  | 36 |
|  | Southeast Asia | Republic of Korea |  | 10 | ≤0.05 | 87 |
|  | Central America | Mexico^‡^ |  | 6 | ≤0.05 | 238 |
|  |  |  |  | 3 |  | 238 |
|  | Southern Asia | Sri Lanka |  | 14 | ≤0.05 | 25 |
|  | Southeast Asia | Vietnam |  | 14 | >0.05 | 50 |
|  | Southern Asia | Sri Lanka |  |  | ≤0.05 | 8 |
|  | Western Pacific | PNG |  | 3 | >0.05 | 108 |
|  |  |  |  | 1 |  | 108 |
|  | Western Pacific | PNG |  | 1 | >0.05 | 1118 |
|  | South America | Brazil |  | 14 | >0.05 | 56 |
|  | South America | Colombia (a) |  | 3 | >0.05 | 42 |
|  |  | Colombia (b) |  |  |  | 16 |
|  | South America | Brazil (a) |  | 3 | >0.05 | 15 |
|  |  | Brazil (a) |  | 8 |  | 15 |
|  |  | Brazil (b) |  | 3 |  | 16 |
|  |  | Brazil (b) |  | 8 |  | 16 |
|  |  | Brazil (c) |  | 3 | ≤0.05 | 11 |
|  |  | Brazil (c) |  | 8 |  | 11 |
|  | Southern Asia | Sri Lanka |  | 2 | ≤0.05 | 386 |
|  | Southeast Asia | Vietnam |  | 1 | >0.05 | 69 |
|  |  |  |  | 14 |  | 69 |
|  |  |  |  | 1 |  | 69 |
|  | South America | Peru (a) |  | 14 | ≤0.05 | 15 |
|  |  | Peru (a) |  | 1 |  | 15 |
|  |  | Peru (b) |  | 14 |  | 9 |
|  |  | Peru (b) |  | 1 |  | 9 |
|  |  | Peru (c) |  | 14 |  | 39 |
|  |  | Peru (c) |  | 1 |  | 39 |
|  |  | Peru (d) |  | 14 |  | 31 |
|  |  | Peru (d) |  | 1 |  | 31 |
|  | Eurasia | Turkey |  | 1 | ≤0.05 | 30 |
|  |  |  |  | 3 |  | 30 |

* Total number of field sites can be concatenated to 35 sites, as there are instances of field site duplication between different studies.

§ Marker family defined as the parent reference for the first generation marker: A, Karunaweera et al. 2007; B, Imwong et al. 2007a; C, Joy et al. 2008; D, Rezende et al. 2010; E, Van den Eede et al. 2010a.

† Total number of microsatellite markers may have been more in published study, but only markers used in more than one field site were considered in this analysis.

‡ Multiple field sites, but endemicity not clearly defined. As a result, all parameters for each site were concatenated into one flat file.

**References**

1. Ferreira MU, Karunaweera ND, da Silva-Nunes M, da Silva NS, Wirth DF, Hartl DL: **Population structure and transmission dynamics of Plasmodium vivax in rural Amazonia.** *J Infect Dis* 2007, **195:**1218-1226.

2. Karunaweera ND, Ferreira MU, Hartl DL, Wirth DF: **Fourteen polymorphic microsatellite DNA markers for the human malaria parasite Plasmodium vivax.** *Molecular Ecology Notes* 2007, **7:**172-175.

3. Gunawardena S, Karunaweera ND, Ferreira MU, Phone-Kyaw M, Pollack RJ, Alifrangis M, Rajakaruna RS, Konradsen F, Amerasinghe PH, Schousboe ML, et al: **Geographic structure of Plasmodium vivax: microsatellite analysis of parasite populations from Sri Lanka, Myanmar, and Ethiopia.** *Am J Trop Med Hyg* 2010, **82:**235-242.

4. Honma H, Kim JY, Palacpac NM, Mita T, Lee W, Horii T, Tanabe K: **Recent increase of genetic diversity in Plasmodium vivax population in the Republic of Korea.** *Malar J* 2011, **10:**257.

5. Imwong M, Nair S, Pukrittayakamee S, Sudimack D, Williams JT, Mayxay M, Newton PN, Kim JR, Nandy A, Osorio L , Carlton JM, White NJ, Day NP, Anderson TJ: **Contrasting genetic structure in Plasmodium vivax populations from Asia and South America.** *Int J Parasitol* 2007, **37:**1013-1022.

6. Imwong M, Pukrittayakamee S, Pongtavornpinyo W, Nakeesathit S, Nair S, Newton P, Nosten F, Anderson TJ, Dondorp A, Day NP, White NJ: **Gene amplification of the multidrug resistance 1 gene of Plasmodium vivax isolates from Thailand, Laos, and Myanmar.** *Antimicrob Agents Chemother* 2008, **52:**2657-2659.

7. Iwagami M, Fukumoto M, Hwang SY, Kim SH, Kho WG, Kano S: **Population structure and transmission dynamics of Plasmodium vivax in the Republic of Korea based on microsatellite DNA analysis.** *PLoS Negl Trop Dis* 2012, **6:**e1592.

8. Joy DA, Gonzalez-Ceron L, Carlton JM, Gueye A, Fay M, McCutchan TF, Su XZ: **Local adaptation and vector-mediated population structure in Plasmodium vivax malaria.** *Mol Biol Evol* 2008, **25:**1245-1252.

9. Karunaweera ND, Ferreira MU, Munasinghe A, Barnwell JW, Collins WE, King CL, Kawamoto F, Hartl DL, Wirth DF: **Extensive microsatellite diversity in the human malaria parasite Plasmodium vivax.** *Gene* 2008, **410:**105-112.

10. Koepfli C, Mueller I, Marfurt J, Goroti M, Sie A, Oa O, Genton B, Beck HP, Felger I: **Evaluation of Plasmodium vivax genotyping markers for molecular monitoring in clinical trials.** *J Infect Dis* 2009, **199:**1074-1080.

11. Koepfli C, Ross A, Kiniboro B, Smith TA, Zimmerman PA, Siba P, Mueller I, Felger I: **Multiplicity and diversity of Plasmodium vivax infections in a highly endemic region in Papua New Guinea.** *PLoS Negl Trop Dis* 2011, **5:**e1424.

12. Orjuela-Sanchez P, da Silva NS, da Silva-Nunes M, Ferreira MU: **Recurrent parasitemias and population dynamics of Plasmodium vivax polymorphisms in rural Amazonia.** *Am J Trop Med Hyg* 2009, **81:**961-968.

13. Restrepo E, Imwong M, Rojas W, Carmona-Fonseca J, Maestre A: **High genetic polymorphism of relapsing P. vivax isolates in northwest Colombia.** *Acta Trop* 2011, **119:**23-29.

14. Rezende AM, Tarazona-Santos E, Fontes CJ, Souza JM, Couto AD, Carvalho LH, Brito CF: **Microsatellite loci: determining the genetic variability of Plasmodium vivax.** *Trop Med Int Health* 2010, **15:**718-726.

15. Schousboe ML, Rajakaruna RS, Amerasinghe PH, Konradsen F, Ord R, Pearce R, Bygbjerg IC, Roper C, Alifrangis M: **Analysis of polymorphisms in the merozoite surface protein-3alpha gene and two microsatellite loci in Sri Lankan Plasmodium vivax: evidence of population substructure in Sri Lanka.** *Am J Trop Med Hyg* 2011, **85:**994-1001.

16. Van den Eede P, Erhart A, Van der Auwera G, Van Overmeir C, Thang ND, Hung le X, Anne J, D'Alessandro U: **High complexity of Plasmodium vivax infections in symptomatic patients from a rural community in central Vietnam detected by microsatellite genotyping.** *Am J Trop Med Hyg* 2010, **82:**223-227.

17. Van den Eede P, Van der Auwera G, Delgado C, Huyse T, Soto-Calle VE, Gamboa D, Grande T, Rodriguez H, Llanos A, Anne J, et al: **Multilocus genotyping reveals high heterogeneity and strong local population structure of the Plasmodium vivax population in the Peruvian Amazon.** *Malar J* 2010, **9:**151.

18. Zeyrek FY, Tachibana S, Yuksel F, Doni N, Palacpac N, Arisue N, Horii T, Coban C, Tanabe K: **Limited polymorphism of the Plasmodium vivax merozoite surface protein 1 gene in isolates from Turkey.** *Am J Trop Med Hyg* 2010, **83:**1230-1237.
